# Clinical Characteristics and Management of Immune Checkpoint Inhibitor-Associated Sicca Syndrome

**DOI:** 10.3390/cancers18111836

**Published:** 2026-06-04

**Authors:** Meridith L. Balbach, Douglas B. Johnson

**Affiliations:** 1 Division of Rheumatology and Immunology, Department of Medicine, Vanderbilt University Medical Center, Nashville, TN 37232, USA; meridith.balbach@vumc.org; 2 Division of Hematology and Oncology, Department of Medicine, Vanderbilt University Medical Center, Nashville, TN 37232, USA

**Keywords:** sicca syndrome, immune checkpoint inhibitors, immune-related adverse events, Sjogren’s disease, xerostomia, immunotherapy

## Abstract

*Background*: Immune checkpoint inhibitors (ICIs) can induce a sicca-like syndrome that differs from primary Sjögren’s disease in both immunopathogenesis and clinical phenotype. Despite growing recognition of this entity, data describing real-world management and outcomes, particularly in the context of ICI discontinuation and rechallenge, remain limited. *Methods*: Patients with new onset of sicca syndrome or exacerbation of previous symptoms following ICI therapy were retrospectively identified and assessed. *Results*: Fifty-nine patients with diverse malignancies (including melanoma, gastrointestinal, genitourinary, etc.) and sicca syndrome following treatment with ICIs (most often pembrolizumab or nivolumab +/− ipilimumab) were evaluated. Acute-onset dry mouth, primarily CTCAE v6.0 grades 1 (n = 24, 40.7%) and 2 (n = 34, 57.6%), occurred at a median of 104 days after ICI initiation, sometimes with associated dry eye (n = 8, 13.6%). Most were managed conservatively with behavioral modification and over-the-counter therapies alone (n = 37, 62.7%) while others received sialagogues (n = 9, 15.3%), dexamethasone oral rinse (n = 11, 18.6%), and/or systemic corticosteroids (n = 16, 27.1%). Additional management strategies included de-escalation to ICI monotherapy (n = 5, 8.5%) or discontinued ICI (n = 6, 10.2%). Half of patients treated with corticosteroids demonstrated subjective improvement in symptoms while 75% improved following ICI discontinuation. Four patients underwent rechallenge after a median interruption of 564 days; all (n = 4) demonstrated sicca recurrence. *Conclusions*: In this largest cohort to date of ICI-associated sicca syndrome, we confirm frequent improvement with steroids and/or supportive care and suggest a greater than previously appreciated risk of recurrence with rechallenge.

## Simple Summary

Cancer immunotherapies have transformed treatment by helping the patient’s immune system attack cancer cells. However, side effects may occur when the immune system attacks healthy tissues. One such side effect is severe dry mouth and eyes (“sicca”), which occurs when moisture-producing glands are affected. In this study, we describe the real-world experiences of patients who developed dry mouth after immunotherapy. Many patients had symptoms that could be managed with supportive treatments such as artificial saliva. Others required medications such as steroids or even stopping immunotherapy altogether. Importantly, we found that symptoms may recur when immunotherapy is restarted, suggesting a higher risk of relapse than was previously recognized. These findings may help guide future treatment decisions for patients experiencing this side effect.

## 1. Introduction

The utility of immune checkpoint inhibitors (ICIs) is evidenced by their expanding clinical application across a variety of cancers and stages. ICIs release the “brakes” on the anti-tumor response through targeted inhibition of negative regulators of T-cell activation such as cytotoxic T-lymphocyte-associated antigen 4 (CTLA4) and programmed cell-death ligand 1 (PD-L1). While this mechanism may augment immune-mediated anti-tumor activity, it may also promote pathologic immune responses to healthy tissue. These unintended targets of toxicity are increasingly recognized as immune-related adverse events (irAEs) [1,2,3]. This recognition has prompted efforts to better characterize the presentation, natural history, and suggested treatment strategies for various irAEs.

One moderately uncommon but clinically significant irAE is ICI-associated sicca syndrome. While xerostomia has been reported in approximately 3–24% of patients receiving ICIs, the true incidence of ICI-associated sicca syndrome remains poorly defined [4]. Several case series have described a clinical phenotype distinct from classical Sjogren’s disease including older age at presentation, male predominance, and absence of typical serologic markers [4,5,6,7,8,9]. Furthermore, these patients demonstrate improved symptoms with systemic corticosteroids and temporary ICI cessation.

Comparative histopathologic analyses of salivary gland biopsies may provide insight into the immunologic mechanisms underlying these phenotypic differences. Warner et al. demonstrated that glands from patients with ICI-associated sicca (n = 20) are characterized by predominant CD8+ T-cell infiltrates, with a relative paucity of CD4+ T-cells and germinal centers, in contrast to the CD4+ T-cell- and plasma cell-rich aggregates typical of Sjogren’s syndrome [4,10]. Similar observations by Brugués et al. (n = 15), Pringle et al. (n = 1), Segawa et al. (n = 1), and Takahashi et al. (n = 1) consistently demonstrate T-cell predominance with sparse B cell involvement [11,12,13,14]. Together, these findings support a cytotoxic, T-cell mediated injury with limited humoral involvement [15].

Given these suspected differences in underlying immunopathogenesis and clinical phenotype, the management of ICI-associated sicca syndrome warrants special consideration. Although there is growing recognition of this entity, current treatment strategies are largely extrapolated from primary Sjogren’s syndrome and general irAE management guidelines [16,17]. However, available data suggests subjective improvement with corticosteroid treatment but limited reversibility of objective salivary flow defects [4,6]. Accordingly, most groups favor a stepwise approach to management, ranging from symptomatic therapies to cautious trials of systemic corticosteroids and, in severe cases, holding ICI therapy [4]. Despite these efforts, optimal treatment strategy remains undefined.

In this report, we add to the growing body of evidence addressing this knowledge gap with the largest cohort to date of patients with a sicca-like syndrome following ICI exposure. We provide a detailed clinical characterization of 59 patients affected by ICI-associated sicca syndrome, with the aim of informing future management strategies and highlighting areas in need of prospective study.

## 2. Materials and Methods

### 2.1. Patient Cohort

**Figure 1:**
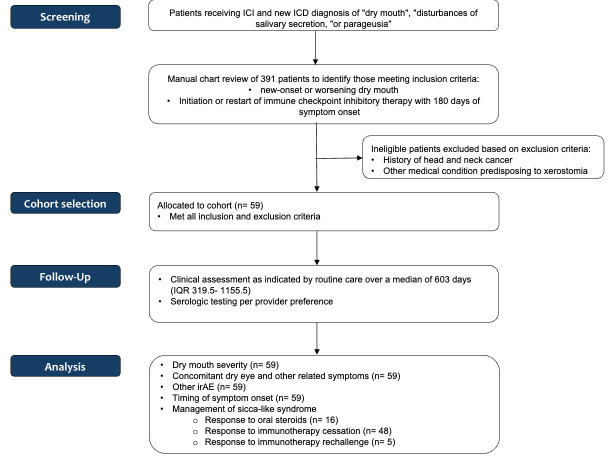
Cohort identification and selection. Patients with new diagnoses of “dry mouth,” “disturbances of salivary secretion,” or “parageusia” following ICI therapy were screened for eligibility. Manual chart review was performed to confirm eligibility based on predefined inclusion and exclusion criteria. A cohort of 59 patients was followed as part of routine clinical care at the discretion of the treating oncologist for a median of 603 days (IQR 320–1156). Clinical characteristics of sicca syndrome, management strategies, and treatment responses were subsequently analyzed. ICI = immune checkpoint inhibitor therapy; irAE = immune-related adverse event; IQR = interquartile range.

We retrospectively screened 386 patients initiating ICI therapy at Vanderbilt University Medical Center from February 2017 to March 2025 through an Institutional Review Board (IRB #150625)-approved protocol (Figure 1). Patients were identified through an electronic medical record search for first-time documentation of the diagnoses “dry mouth,” “disturbances of salivary secretion,” or “parageusia” following ICI initiation. Manual chart review was subsequently performed to ascertain symptom attribution and cohort eligibility. Inclusion criteria were defined as (1) new-onset or worsening dry mouth (2) following initiation or restart of ICI therapy, and (3) in the absence of other likely alternative etiology of xerostomia. Patients initiating medications with a known dry mouth side effect (such as opioids, antihistamines, antidepressants, etc.) within 30 days of sicca onset were excluded. Patients with head and neck cancer or other confounding medical conditions predisposing to xerostomia were excluded due to difficulty ascertaining the cause of xerostomia. Baseline patient characteristics including age, sex, smoking status, BMI, and pre-existing autoimmune disease were recorded. No patients underwent salivary gland biopsy.

### 2.2. Outcome Measures

Dry mouth severity was graded according to the Common Terminology Criteria for Adverse Events (CTCAE, version 6.0) based on documented patient-reported symptoms and clinician assessment. Objective salivary gland measures (such as salivary flow) were not performed. Concomitant dry eye and mucositis were also assessed based on subjective measures. Treatment strategies were recorded. Time from ICI initiation to sicca onset was defined as the interval between initiation of ICI therapy and development of new sicca symptoms. In patients with pre-existing dry mouth, sicca onset was defined as the interval between ICI initiation and worsening dry mouth attributed to ICI therapy. Response to therapy, immunotherapy cessation, and rechallenge (if applicable) was assessed as “no change”, “improvement”, or “resolution” based on clinician documentation of patient-reported outcomes during subsequent follow-up visit(s). Immunologic laboratory data (ANA, SSA/SSB, rheumatoid factor), if obtained, were assessed. Descriptive statistics were expressed using median and interquartile range (IQR; 25th–75th percentile). Normality of continuous variables was assessed using the Shapiro–Wilk test. Post hoc exploratory analyses used the Wilcoxon rank-sum test for continuous variables and Fisher’s exact test for categorical variables. Where applicable, categorical data were dichotomized (i.e., CTCAE severity: grade 1 = “low severity” and grade 2 or higher = “high severity”; response to ICI discontinuation: no improvement “not responsive” and improvement or resolution = “responsive”).

## 3. Results

### 3.1. Patient Characteristics

A cohort consisting of 59 patients meeting all inclusion and exclusion criteria was identified. Baseline patient characteristics of this expanded cohort were similar to those previously reported, including 33 men and 26 women with a median age of 67 years (IQR 54.3–73) at time of ICI initiation (Table 1). A variety of cancer types were represented, including melanoma (n = 18), gastrointestinal (n = 15; including cholangiocarcinoma and hepatocellular carcinoma), genitourinary (n = 13; including renal cell carcinoma and urothelial carcinoma), and others. Most (n = 47) patients had no known prior history of autoimmune disease although several had hypothyroidism (n = 10), rheumatoid arthritis (n = 2), and/or psoriasis (n = 1). Most patients received combination therapy with ipilimumab and nivolumab (n = 19) or monotherapy with pembrolizumab (n = 17), or nivolumab (n = 9).

### 3.2. Presentation of ICI-Associated Sicca Syndrome

Fifty-three (89.8%) patients reported new-onset dry mouth while six (10.2%) reported noticeable worsening of pre-existing dry mouth following ICI exposure. A range of severity was appreciated, with most CTCAE v6.0 grade 1 (n = 24, 40.7%) or grade 2 severity requiring dietary modification (n = 34, 57.6%). One patient developed grade 4 toxicity requiring ICU admission for IV fluid resuscitation for severe hyponatremia in the setting of sicca-related poor oral intake (Supplementary S2). Subjectively, most patients reported an acute onset, particularly in those with a greater severity of symptoms. Many noted gradual worsening while on therapy. Associated symptoms included taste disturbance, oral pain, and weight loss. Several (n = 8, 13.6%) also developed CTCAE v6.0 grade 1 dry eye.

The median time to symptom onset from initiation of ICI therapy was 104 days (IQR 51.5–159.5). Patients were followed for a median of 603 days (IQR 319.5–1155.5) from sicca onset. Most (n = 43, 72.9%) patients did not undergo serologic testing (see Supplementary S1). Of those who did, most (n = 6, 10.2%) were negative for both anti-SSA/Ro or anti-SSB/La while one patient tested weakly positive for anti-SSB/La at 1.1 (upper limit of normal 0.91) but negative for anti-SSA/Ro. Of patients tested for antinuclear antibodies (n = 11, 18.6%), about half (n = 6) were either weakly (n = 2) or moderately (n = 4) positive. Most (n = 41, 69.5%) patients demonstrated one or more additional irAEs including thyroiditis (n = 18, 30.5%), cutaneous toxicity (n = 15, 25.4%), colitis (n = 9, 15.3%), hepatitis (n = 6, 10.2%), arthritis (n = 6, 10.2%), and pneumonitis (n = 4, 6.7%). Many (n = 32, 54.2%) patients were prescribed medications with a possible side effect of dry mouth including opioids (n = 14, 23.7%), antihistamines (n = 13, 22%), antidepressants (n = 12, 20.3%), or other (n = 3, 5.1%), although given temporality, it was felt that ICI therapy was the proximate cause of patient symptoms.

### 3.3. Management of ICI-Associated Sicca Syndrome and Other irAE

Most (n = 37, 62.7%) patients were managed conservatively with behavioral modification (e.g., increased oral hydration, use of sugar-free candies or saliva stimulants) and over-the-counter medications such as artificial saliva, artificial tears, and biotene formulations (spray, gel, or mouth rinse; Table 2). An additional cohort of patients with poor response to first-line management received steroid mouthwash rinse (n = 11, 18.6%) and/or sialagogues (n = 9, 15.3%). Six patients (10.2%) were treated with oral steroid taper due to sicca syndrome while ten (16.9%) were treated with steroid and/or other biologics due to other irAE.

Half (n = 8) of those treated with oral steroids demonstrated improvement in sicca symptoms though without resolution, regardless of initial indication. Among six patients who received oral steroids due to sicca, most (n = 5, 83.3%) demonstrated improvement in dry mouth. Six patients (10.2%) discontinued further immunotherapy due to sicca symptoms while thirteen others stopped due to other irAE, most often pneumonitis (n = 3) or hepatitis (n = 4). Eleven (18.6%) patients switched lines of therapy due to progression of disease. Change in sicca symptoms after immunotherapy cessation was unable to be observed in some patients (n = 11, 18.6%) due to limited follow-up or subsequent death. Of those patients assessed (n = 48, 81.4%), most demonstrated resolution (n = 21, 43.8%) or improvement (n = 15, 31.3%) in sicca, but a significant cohort (n = 12, 25%) demonstrated persistent symptoms without substantial improvement. Of those assessed patients who discontinued immunotherapy due to sicca, all demonstrated resolution (n = 3, 60%) or improvement (n = 2, 40%) in their symptoms.

**Figure 2:**
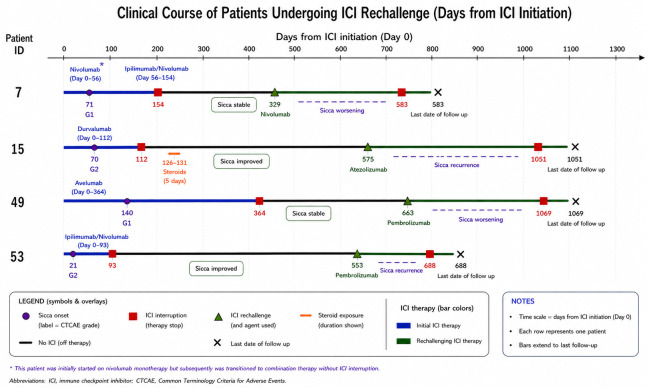
Clinical course of patients with ICI-associated sicca syndrome undergoing ICI rechallenge. Swimmer plot demonstrates timing of sicca onset, ICI interruption, symptom course, and ICI rechallenge among four patients with ICI-associated sicca syndrome. Time is displayed as days from ICI initiation (day zero). See figure legend. Patients 7 and 49 demonstrated stable sicca symptoms after initial ICI discontinuation followed by worsening of symptoms with ICI rechallenge. Patients 15 and 49 demonstrated significant improvement in sicca symptoms after initial ICI discontinuation followed by recurrence of symptoms with ICI rechallenge. * *Patient 7 initially received* 2 cycles of nivolumab and subsequently was transitioned to combination therapy with ipilimumab/nivolumab without interruption. ICI, immune checkpoint inhibitor; CTCAE, Common Terminology Criteria for Adverse Events.

Five (8.5%) patients were rechallenged with either de-escalation from combination to single agent immunotherapy (n = 4, 80%) or transition of agent from nivolumab to pembrolizumab (n = 1, 20%) after a median of 564 days of immunotherapy interruption. Response to ICI rechallenge was unable to be assessed in one patient due to subsequent death. Prior to rechallenge, sicca symptoms had significantly improved (patients 15 and 53) or stably persisted (patients 7 and 49). All evaluable (n = 4) patients developed recurrence or worsening of sicca symptoms with rechallenge (see Figure 2).

We then assessed whether clinical factors were associated with severity or improvement of symptoms. Concomitant use of xerogenic medications was not associated with sicca severity (OR 1.57; *p* = 0.583), suggesting such medications were less likely to confound observations. There was no significant association between occurrence of other irAE and severity of dry mouth (OR = 1.25; *p* = 0.777). However, patients with greater sicca severity were significantly more likely to demonstrate earlier symptom onset than those patients with grade 1 sicca (median 70 versus 139 days; *p*-value = 0.0247). Patients receiving combination therapy were more likely to demonstrate sicca with earlier onset (63 [range 42–128] versus 116 [range 65–170] days, *p* = 0.024) and grade 2 or higher severity (OR 10.0; *p* = 0.001). Receipt of systemic corticosteroids was not associated with a higher likelihood of improvement or resolution of sicca symptoms after immune checkpoint inhibitor discontinuation (OR 1.5; *p* = 0. 75).

## 4. Discussion

In this largest cohort to date of patients with ICI-associated sicca syndrome (n = 59), our findings corroborate those of prior descriptions of four patients by Cappelli et al. in 2017 and 20 patients by Warner et al. in 2020 [4,5]. Like other groups, we observed an acute-onset xerostomia occurring at a median of approximately 3 months after initial ICI exposure, oftentimes with associated xerophthalmia (13.5%) and other extra-glandular irAEs. Whereas Warner et al. described a cohort largely enriched for grade 2 sicca severity, our study captured a broader distribution of disease severity, with nearly half of patients presenting with grade 1 symptoms. Notably, patients receiving combination ICI therapy were substantially more likely to develop higher grade sicca.

Consistent with proposed management guidelines, we observed a stepwise approach to treatment, with more than half of all patients managed with symptomatic therapies alone. Among those treated with systemic corticosteroids, we observed greater variability in subjective symptom improvement (50%) versus Warner et al. (83%) and Greenwood et al. (87.5%), highlighting the heterogeneity of treatment responsiveness in this population [4,6]. Interestingly, 83.3% of patients treated with systemic corticosteroids specifically for sicca symptoms (rather than for other concomitant irAEs) reported symptomatic improvement despite generally demonstrating greater symptom severity. Given the association between greater sicca severity and earlier symptom in our cohort, these observations raise the possibility that earlier-onset presentations may represent a more treatment-responsive phase preceding irreversible glandular dysfunction. Although a substantial proportion of patients (25%) demonstrated persistent sicca symptoms, most reported at least partial subjective improvement following ICI cessation. In contrast to prior findings by Warner et al., in which six of seven patients tolerated ICI rechallenge without recurrence of sicca symptoms, all four rechallenged patients in our cohort demonstrated sicca recurrence, suggesting a greater risk than was previously appreciated [4]. Additional data will be needed to clarify this risk given the small sample size.

While our cohort provides primarily clinical and descriptive data rather than detailed immunophenotypic characterization, these findings are consistent with prior work suggesting a distinct pathogenesis of ICI-associated sicca. In particular, they align with the proposed CD8+ T-cell-mediated cytotoxic model of glandular injury [4,11]. ICIs disrupt key pathways of immune tolerance that are hijacked and co-opted by cancer cells, which may result in autoimmune-like symptoms. Although the factors predisposing some patients to irAEs remain incompletely understood, hypothesized mechanisms include genetic human leukocyte antigen variation, smoldering pre-existing autoimmunity, gut microbiome dysbiosis, or tumor antigen mimicry [18,19,20,21,22]. Immunogenic cell death induced by ICIs may further amplify these processes through the release of damage-associated molecular patterns and tumor antigens, thereby promoting immune responses that may extend beyond the tumor microenvironment [23,24]. Although ICI-associated sicca does not align to a clear mechanistic subset, predictive biomarkers (e.g., autoantibodies, specific T-cell clones) may be useful to identify patients at greatest risk [25].

**Figure 3:**
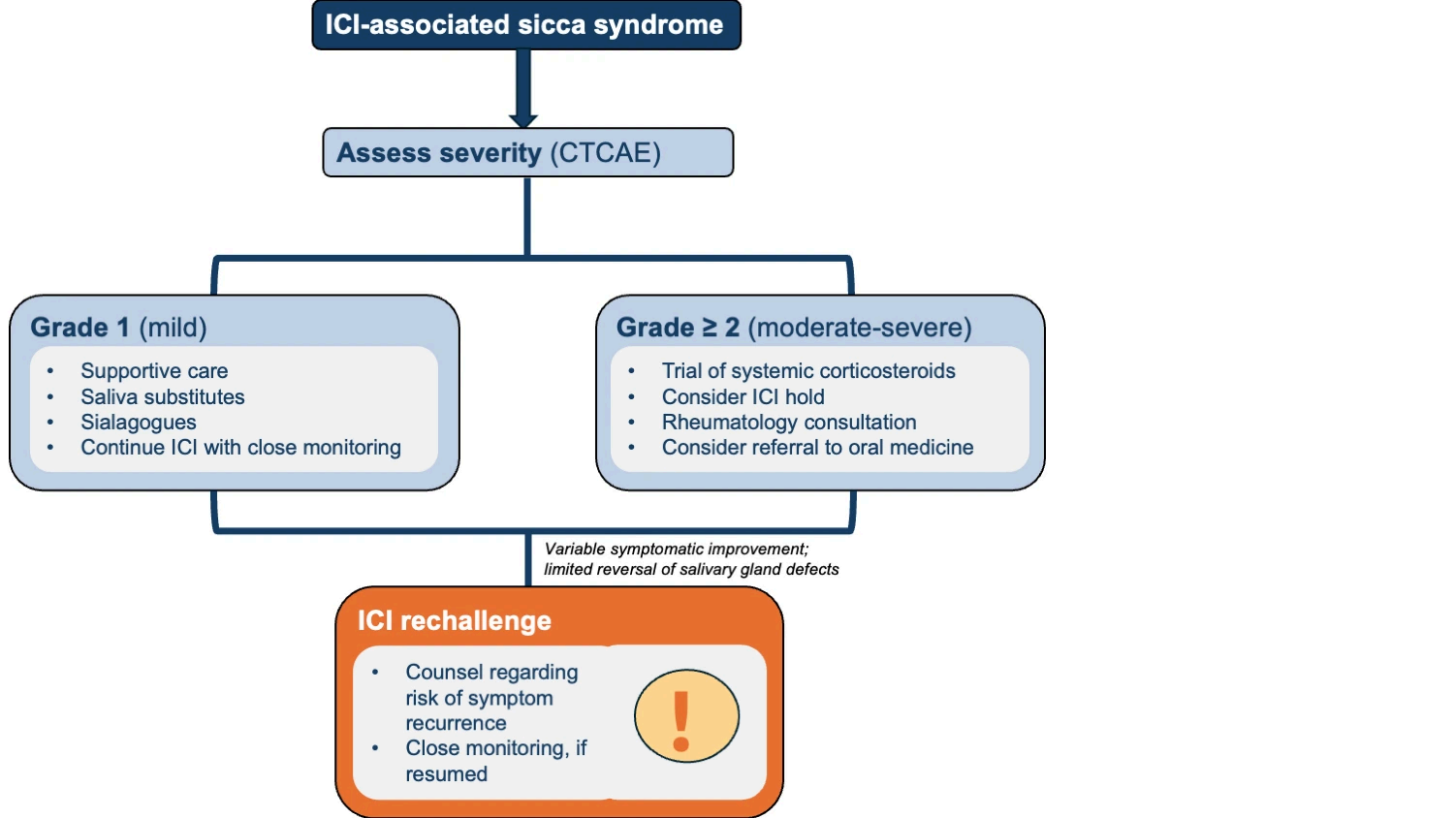
Proposed management framework for ICI–associated sicca syndrome. We suggest a severity-based approach to management informed by the prior literature and the findings of this retrospective study. Initial treatment emphasizes symptomatic-based supportive care for mild disease with consideration of systemic corticosteroids and ICI interruption for moderate to severe symptoms. Risk-benefit of ICI rechallenge should be discussed thoroughly given high risk of symptom recurrence.

Variable responsiveness to corticosteroids and ICI cessation may reflect differences in symptom onset and intervention relative to irreversible glandular injury, in contrast to other irAE endocrinopathies characterized by permanent tissue loss. Consistent with this hypothesis, patients in our study with earlier symptom onset were significantly more likely to demonstrate improvement or resolution of sicca following ICI cessation. Although the most appropriate management strategy remains uncertain, we propose an adapted framework based on the prior literature and emphasizing the risk of recurrence with ICI rechallenge informed by our retrospective observation (Figure 3). However, this hypothesis-generating framework should be used carefully in the absence of evidence-based guidance.

Our findings underscore a limited therapeutic arsenal with notable tradeoffs. Glucocorticoids, while sometimes effective, carry a substantial side effect burden and, particularly when used at higher doses or early in the treatment course, may compromise the clinical benefit of ICI therapy [26,27]. Likewise, ICI cessation, although frequently effective, may not be feasible for patients with limited alternative cancer-directed therapies. These constraints highlight a need for additional management strategies [28]. Notably, there is no available data evaluating the efficacy of non-steroidal anti-inflammatory drugs in ICI-associated sicca, though extrapolation from other rheumatic irAEs such as inflammatory arthritis would suggest limited utility [29]. Similarly, biologics have not been studied in this context. Intriguingly, preclinical data suggest a potential role for IL-17 blockade, with evidence of restored salivary function in a mouse model [30]. However, clinical applicability remains uncertain. Taken together, these limitations in current management emphasize the need to shift from a reactionary therapeutic approach to one that focuses on prevention, early detection, and longitudinal monitoring of at-risk patients.

These results must be interpreted cautiously given several limitations including the retrospective study design, lack of objective outcome measures, inclusion of patients with both de novo sicca and exacerbation of pre-existing symptoms, and a relatively limited sample size for rechallenge evaluation. Furthermore, variable indications for corticosteroid usage may confound assessment of treatment responsiveness. Future prospective studies incorporating objective salivary gland assessments (such as whole unstimulated salivary flow) and standardized outcome measures will be important to better define treatment response and recurrence risk. Further evaluation of treatment responsiveness stratified by disease severity (grade 1 vs. grade 2), timing of intervention onset, and clinicopathologic differences between patients who do and do not respond to corticosteroids and/or ICI discontinuation may help clarify observed heterogeneity while providing additional insight into underlying immunopathogenesis.

## 5. Conclusions

Despite these limitations, our cohort suggests that ICI-associated sicca syndrome represents a distinct rheumatologic irAE with heterogeneous treatment responsiveness. Our findings largely reinforce the treatment framework proposed by Warner et al. while highlighting a need for clearer guidance regarding ICI rechallenge in patients with ICI-associated sicca syndrome.

## Figures and Tables

**Table 1:** Patient baseline characteristics and sicca syndrome features. * One patient demonstrated grade 4 toxicity.

		Years
**Age at time of ICI exposure**	Median	66.5
	IQR	54.3–73
**Sex**		**n (%)**
	Male	33 (55.9)
	Female	26 (44.1)
**Pre-existing autoimmune disease**		**n (%)**
	Hypothyroidism	10 (16.9)
	Rheumatoid arthritis	2 (3.3)
	Psoriasis	1 (1.7)
**Malignancy**		**n (%)**
	Melanoma	18 (30.5)
	Gastrointestinal	15 (25.4)
	Genitourinary	13 (22)
	Lung	10 (16.9)
	Breast	2 (3.3)
	Leiomyosarcoma	1 (1.7)
**ICI agent**		**n (%)**
	Ipilimumab/nivolumab	19 (32.2)
	Pembrolizumab	18 (30.5)
	Nivolumab	9 (15.3)
	Durvalumab	6 (10.2)
	Atezolizumab	6 (10.2)
	Avelumab	1 (1.7)
**Sicca symptom onset**		**n (%)**
	New	53 (89.8)
	Exacerbation	6 (10.2)
**Time from ICI initiation to sicca onset**		**Days**
	Median	104
	IQR	51.5–159.5
**Greatest dry mouth severity, CTAE grade**		**n (%)**
	1	24 (40.1)
	2	34 (57.6)
	3–5	1 * (1.7)
**Greatest dry eye severity,** **CTCAE grade**		**n (%)**
	Not present	51 (86.4)
	1	8 (13.6)
**Other irAE**		**n (%)**
	None	18 (30.5)
	Thyroiditis	18 (30.5)
	Cutaneous	15 (25.4)
	Colitis	9 (15.3)
	Hepatitis	6 (10.2)
	Arthritis	6 (10.2)
	Pneumonitis	4 (6.8)

**Table 2:** Patient management strategies and treatment responses. * Conservative management defined as behavioral modification and over-the-counter medication only.

**Sicca syndrome management (n = 59)**		**n (%)**
	Conservative management only *	37 (62.3)
	Sialagogue	9 (15.3)
	Oral corticosteroid rinse	11 (18.6)
	Systemic corticosteroid (due to sicca)	6 (10.2)
	Systemic corticosteroid (due to other irAE)	10 (16.9)
	Drop 1 line of ICI therapy	5 (8.5)
	Discontinue ICI (due to sicca)	6 (10.2)
**Response to systemic corticosteroids (n = 16)**		**n (%)**
	No change	8 (50)
	Improvement	8 (50)
	Resolution	0
**Response to ICI discontinuation (n = 48)**		**n (%)**
	No change	12 (25)
	Improvement	15 (31.2)
	Resolution	21 (43.8)
	Ongoing active treatment	4
	Lost to follow-up and/or death	7
**ICI rechallenge (n = 4)**		**Days**
Time off therapy	Median	564
	Range	329–663
Rechallenge agent		**n (%)**
	Nivolumab	2 (40)
	Pembrolizumab	2 (40)
	Atezolizumab	1 (20)
Recurrence (n = 4)		**n (%)**
	Recurrent sicca	4 (100)
	Lost to follow-up and/or death	1

## Data Availability

Datasets are available upon reasonable request to the corresponding author.

## Supplementary Materials

The following supporting information can be downloaded at: https://www.mdpi.com/article/10.3390/cancers18111836/s1, S1: Diagnostic workup: This table provides additional patient-level information regarding serologic testing performed (if any) and presence of concomitant medication with dry mouth side effect. No patients received a parotid gland biopsy. Referral to rheumatology, ophthalmology, and/or oral medicine was not recorded. S2: Single case of grade 4 xerostomia.
