# Supplementary material for: Clinical Characteristics and Management of Immune Checkpoint Inhibitor-Associated Sicca Syndrome
Source: Cancers (Basel). 2026 Jun 4;18(11):1836. doi: 10.3390/cancers18111836 (PMC13256113; doi:10.3390/cancers18111836)
Supplement: Supplementary file 1 [file cancers-18-01836-s001.zip › cancers-4277790-supplementary.pdf]

S1 Diagnostic workup: This table provides additional patient-level information regarding serologic testing performed (if any) and presence of concomitant medication with dry mouth side effect. No patients received a parotid gland biopsy. Referral to rheumatology, ophthalmology, and/or oral medicine was not recorded.

Patient ID	Serologic testing	Concomitant xerogenic medications (without concurrent start)
1	None performed	None
2	None performed	Opioid, antihistamine, antidepressant
3	None performed	Antidepressant
4	None performed	None
5	None performed	Antihistamine, antidepressant
6	SSA/SSB negative	None
7	+ANA 1/80; negative extracted nuclear antigen panel	Antihistamine
8	None performed	Opioid
9	None performed	None
10	SSB mildly positive 1.11; SSA negative	Opioid
11	None performed	Antihistamine
12	None performed	Opioid, antihistamine
13	None performed	None
14	None performed	Opioid; other anticholinergic
15	SSA/SSB negative	None
16	None performed	None
17	None performed	None
18	ANA+ 1/320; SSA-, SSB-	None
19	None performed	None
20	None performed	Other cancer-directed therapy
21	None performed	Antihistamine
22	None performed	Opioid
23	None performed	None
24	None performed	None
25	None performed	None
26	None performed	None
27	ANA 1/320; negative rheumatoid factor; CRP 69; ESR 31	Antidepressant
28	None performed	None
29	None performed	Antidepressant
30	None performed	None
31	SSA/SSB negative-; ANA 1/160	Antihistamine
32	None performed	Antidepressant
33	None performed	None

34	None performed	None
35	None performed	Antihistamine
36	None performed	Opioid, antihistamine, antidepressant
37	Negative SSA/SSB; Negative ANA	Opioid
38	None performed	Opioid, antidepressant
39	None performed	Antihistamine
40	None performed	Antihistamine
41	None performed	None
42	None performed	None
43	None performed	Antihistamine, antidepressant
44	Negative SSA/SSB; +ANA 1/320 with negative extractable nculear antigen panel	Antidepressant
45	None performed	Opioid
46	Negative ANA	Opioid
47	None performed	Antidepressant
48	None performed	Opioid
49	None performed	None
50	Negative ANA	None
51	None performed	None
52	None performed	None
53	None performed	None
54	Negative ANA	Opioid
55	None performed	None
56	ANA+ (1/40)	Antidepressant
57	Negative ANA	Antihistamine
58	Negative ANA	None
59	Negative rheumatoid factor	Other cancer-directed therapy

S2: Single case of grade 4 xerostomia

Patient 44 developed grade 4 xerostomia thought to be ICI-associated sicca. New dry mouth symptoms were noted 168 days after ICI initiation. This progressed over several weeks and patient reported significant increase in free water uptake to alleviate dry mouth symptoms. Simultaneously, she decreased solid food intake due to pain associated with eating. She was found to be hyponatremic to 120 and required inpatient admission for intravenous fluid resuscitation. She improved with fluid resuscitation, free water restriction and oral prednisone taper.
